# Effects of elevated CO_2_ concentration and experimental warming on morphological, physiological, and biochemical responses of winter wheat under soil water deficiency

**DOI:** 10.3389/fpls.2023.1227286

**Published:** 2023-08-04

**Authors:** Zhijie Chang, Lihua Hao, Yunze Lu, Liang Liu, Changhua Chen, Wei Shi, Yue Li, Yanrui Wang, Yinshuai Tian

**Affiliations:** ^1^ School of Water Conservancy and Hydropower, Hebei University of Engineering, Handan, Hebei, China; ^2^ School of Landscape and Ecological Engineering, Hebei University of Engineering, Handan, Hebei, China; ^3^ School of Earth Science and Engineering, Hebei University of Engineering, Handan, Hebei, China

**Keywords:** water deficiency, CO_2_, warming, biomass, photosynthesis, gene expression

## Abstract

Global climate change and freshwater scarcity have become two major environmental issues that constrain the sustainable development of the world economy. Climate warming caused by increasing atmospheric CO_2_ concentration can change global/regional rainfall patterns, leading to uneven global seasonal precipitation distribution and frequent regional extreme drought events, resulting in a drastic reduction of available water resources during the critical crop reproduction period, thus causing many important food-producing regions to face severe water deficiency problems. Understanding the potential processes and mechanisms of crops in response to elevated CO_2_ concentration and temperature under soil water deficiency may further shed lights on the potential risks of climate change on the primary productivity and grain yield of agriculture. We examined the effects of elevated CO_2_ concentration (*e*[CO_2_]) and temperature (experimental warming) on plant biomass and leaf area, stomatal morphology and distribution, leaf gas exchange and mesophyll anatomy, rubisco activity and gene expression level of winter wheat grown at soil water deficiency with environmental growth chambers. We found that *e*[CO_2_] × water × warming sharply reduced plant biomass by 57% and leaf photosynthesis (*P*_n_) 50%, although elevated [CO_2_] could alleviated the stress from water × warming at the amount of gene expression in *RbcL3* (128%) and *RbcS2* (215%). At ambient [CO_2_], the combined stress of warming and water deficiency resulted in a significant decrease in biomass (52%), leaf area (50%), *P*_n_ (71%), and *G*_s_ (90%) of winter wheat. Furthermore, the total nonstructural carbohydrates were accumulated 10% and 27% and increased *R*_d_ by 127% and 99% when subjected to water × warming and *e*[CO_2_] × water × warming. These results suggest that water × warming may cause irreversible damage in winter wheat and thus the effect of “CO_2_ fertilization effect” may be overestimated by the current process-based ecological model.

## Introduction

Atmospheric CO_2_ concentration ([CO_2_]) has currently exceeded 420 *µ*mol mol^−1^ with an increase rate of 2 *µ*mol mol^−1^ from 280 *µ*mol mol^−1^ at the beginning of the industrialization era and will continually be over 800 *µ*mol mol^−1^ by the end of 21^st^ century (IPCC, 2021). As a result, the increased atmospheric [CO_2_] has resulted in a rapid rise in global surface temperature over the past decades (Barlow et al., 2015; Jin et al., 2017), even the average global temperature is predicted to rise 2 to 6 °C depending on the concentrations of greenhouse gases such as CO_2_ in the atmosphere (IPCC, 2021). It has been estimated that global precipitation may decline under future climatic warming, and meanwhile the spatial and temporal patterns of global/regional precipitation distribution may also become uneven (Chun et al., 2011; Zhang et al., 2020), thus drought and elevated temperature may constrain plant growth and crop yield alone or in combination (Yu et al., 2012), although elevated [CO_2_] generally facilitates plant growth and promotes plant adaptation to climate change (Kirkham, 2011; Xu, 2015; Li et al., 2021). Unfortunately, simultaneous drought stress, elevated [CO_2_] and elevated temperature has already occurred in summer in many regions throughout the world, which is even more detrimental to plant growth than either stress alone. Nevertheless, the underlying response mechanisms of elevated [CO_2_] and elevated temperature on crops such as winter wheat at different soil water status are still unclear (Abebe et al., 2016; Fan et al., 2020; Zheng et al., 2020), and thus these uncertainties not only restrict the capacity to accurately predict agricultural carbon sequestration, but also limit understanding of the potential impacts of climate change on agricultural productivity (Ainsworth and Long, 2020; Muluneh, 2020).

It has been well demonstrated that various physiological, biochemical, and molecular processes of plants are sensitive to water deficiency, elevated [CO_2_], and elevated temperature alone or the combination (Mirwais et al., 2006; Yu et al., 2012; Bencze et al., 2014; Xu, 2015; Zheng et al., 2018; Duan et al., 2019a; Zheng et al., 2020; Li et al., 2021). Elevated [CO_2_] generally promotes leaf photosynthesis, plant growth, and crop yield through the “CO_2_ fertilization” effect with enhancing the ribulose-1,5-bisphosphate carboxylase oxygenase (Rubisco) carboxylation efficiency (Arndal et al., 2014; Niaz et al., 2020) and inhibiting leaf respiration rates (Yu et al., 2012; Xu, 2015). For example, Duan et al. (2019a) found that elevated [CO_2_] from 400 *μ*mol mol^−1^ to 640 *μ*mol mol^−1^ increased plant biomass, leaf area, and leaf number of *Eucalyptus tereticornis*, which attributed to the enhanced net photosynthetic rate and the reduced dark respiration. However, several recent studies have reported that soil water status could modify the CO_2_ fertilization effect on crops such as winter wheat (Zheng et al., 2020) and green pepper (Fan et al., 2020) with changing leaf photosynthesis, stomatal traits, and non-structural carbohydrates, which are generally regulated by soil water availability and plant water status (Yu et al., 2012; Duan et al., 2019a). On the other hand, water deficiency often results in obvious reduction in leaf photosynthesis and plant biomass, and elevated [CO_2_] may mitigate the negative effects of water deficiency with regulating leaf photosynthesis, stomatal conductance, and leaf transpiration (Fan et al., 2020), which are coupled with stomatal traits such as stomatal density, stomatal openness, and the distribution pattern of stomata (Xu, 2015; Zheng et al., 2020). Nevertheless, the underlying mechanisms and processes of elevated [CO_2_] alleviates the effect of water stress on crops is remain unknown, thus studies focus on these topics will help to fill the knowledge gap, and meanwhile allow us to fully understand the impacts of water stress on global agriculture productivity in a higher atmospheric [CO_2_] world.

Due to the elevated atmospheric [CO_2_], climate models have also projected that water deficiency may also be accompanied by elevated temperature, namely climate warming. It is well demonstrated that many physiological processes of plants such as leaf photosynthesis and respiration are strongly dependent on growth temperature and soil/plant water status (Hatfield et al., 2011; Zhang et al., 2015). As a result, elevated temperature and drought stress usually decrease leaf photosynthesis and increase leaf respiration (Parry et al., 2003; Kurek et al., 2007; Bencze et al., 2014; Duan et al., 2019b), and limit plant growth and reduce crop yields when growth temperature and soil water content exceed the optimum for plant growth (Salvucci and Crafts-Brandner, 2004; Zhao et al., 2017; Hao et al., 2019). In addition, the negative effects of water deficiency and heat stress on plant growth and crop yield can be mitigated with elevated [CO_2_] (Mirwais et al., 2006; Wei et al., 2018). Mirwais et al. (2006) show that higher temperatures or drought inhibited many processes, while elevated [CO_2_] partially mitigated some of the adverse effects, such as total dry matter mass, net photosynthetic rate, and abscisic acid. Many previous studies have investigated the mitigating effects of elevated [CO_2_] on crop drought or warming, but the effect of high [CO_2_] on crop leaf structure, physiological processes, and biochemical synergistic responses to water deficiency and warming has rarely been reported (Mirwais et al., 2006; Yu et al., 2012).

Winter wheat (*Triticum aestivum* L.) in the North China Plain accounts for about 40% of the total arable land area and is one of the most important foodstuffs for humans (Wang et al., 2016). However, previous work predicted that the global climate has warmed in recent years and that groundwater resources are scarce (Tao et al., 2006), so not only warming and water deficiency may directly affect winter wheat growth and development by altering physiological and biochemical processes (Farooq et al., 2011; Zheng et al., 2020), but water deficiency and warming may also indirectly and synergistically affect grain yield of winter wheat as crop phenology and growth stages change (Hatfield et al., 2011). Wall et al. (2006) has shown that elevated [CO_2_] can increase the net photosynthetic rate in winter wheat, alleviate warming or water deficiency by reducing stomatal conductance and transpiration rate, and improve crop water use efficiency (Keshav et al., 2014). Meanwhile, elevated [CO_2_] can also inhibit the respiration process and Rubisco oxygenation reaction in winter wheat, which in turn increases the net photosynthetic rate (Li et al., 2004; Xu, 2015). Understanding the impact of elevated [CO_2_] on the physiology of winter wheat in response to water deficiency and/or warming alone or simultaneously has great potential for the development of stress-tolerant germplasm and new practices. Therefore, the objectives of this study were to observe (1) the interactive effect of elevated [CO_2_], experimental warming, and water deficiency on morphological traits and physiological processes; (2) the relationships between gas exchange, stomatal characteristics, leaf anatomical characteristics, non-structural carbohydrates, and Rubisco gene expression under *e*[CO_2_] × water × warming; and (3) to investigate whether elevated [CO_2_] could mitigate the negative effects of water deficiency or warming on winter wheat.

## Materials and methods

### Plant materials and experimental design

In the current study, a commonly cultivated variety (Shimai 15) of winter wheat (*Triticum aestivum*) in the North China Plain was selected as the research material. We firstly sowed six wheat seeds in each plastic pot, where the height was 27 cm and the top and bottom areas were 531 cm^2^ and 380 cm^2^, respectively. This large volume at the bottom of pot was enough for root growth of winter wheat plants. The soil in pots for sustaining plant growth was the mixtures of yellow loam and nutrient soil with a volume ratio of 2:1. In addition, the field capacity was 24.5% and the bulk density was 1.28 g cm^-3^ of soil. Then, four pots were set up to each of eight environmental growth chambers (Model BDP-2000, Ningbo Prandt Instrument Co., Ltd, China) to sustain plant growth with a temperature regime of 21/16°C (day/night) and 1000 *μ*mol m^−2^ s^−1^ photosynthetic active radiation, and 60%-70% relative humidity for 30 days for establishing plant canopy and roots. The space inside these environmental growth chambers (1.83 m high × 1.79 m long × 0.68 m wide) was large enough for the growth of winter wheat.

Wheat plants were treated with a split plot design consisting of three environmental factors (water, CO_2_ concentration, and temperature), where watering was treated as the main plot, and temperature and CO_2_ concentration were treated as subplots. Therefore, wheat plants in the eight environmental growth chambers were subjected to two water conditions, namely plants in four environmental growth chambers were well-watered with 75%-80% field capacity as full irrigation, and plants in the other four environmental growth chambers were treated as water deficiency with 45%-50% field capacity. The soil water content in all pots were measured with a Time Domain Reflectometry (TDR). The CO_2_ concentrations were controlled at two levels of 400 *μ*mol mol^−1^ (ambient CO_2_ concentration, *a*[CO_2_]) and 800 *μ*mol mol^−1^ (elevated CO_2_ concentration, *e*[CO_2_]), and growth temperature treatments were 21/16°C (day/night, optimal growth temperature) and 26/21°C (day/night, elevated growth temperature). Therefore, the four environmental growth chambers subjected to full irrigation or water deficiency were randomly allocated to four treatments including Control (growth temperature is 21/16°C and CO_2_ concentration is 400 *µ*mol mol^−1^), *e*[CO_2_] (growth temperature is 21/16°C and CO_2_ concentration is 800 *µ*mol mol^−1^), Warming (growth temperature is 26/21°C and CO_2_ concentration is 400 *µ*mol mol^−1^), and *e*[CO_2_] × warming (growth temperature is 26/21°C and CO_2_ concentration is 800 *µ*mol mol^−1^). In the current study, the four pots planted with winter wheats in each environmental growth chamber were treated as the biological replicates (n=4). The photosynthetic active radiation was 1000 *μ*mol m^−2^ s^−1^ with a 12-h photoperiod from 8:00 to 20:00, and relative humidity was controlled at 60%-70% in the eight environmental growth chambers. Wheat plants were fertilized once a week with half-strength Hoagland solution during the 90-day treatment period. Additionally, in the current study, we randomly changed the [CO_2_] of each growth chamber to reduce the confounding effects from the various environmental growth chambers while concurrently shifting the treated winter wheat to the environmental growth chambers with appropriate [CO_2_] monthly.

### Measurement on leaf area, plant biomass, and leaf anatomy

All leaves of the winter wheat from each pot were sampled and leaf area was measured with a leaf area meter (LI-3100, LICOR, USA). Then, all the winter wheat tissues (leaves, stems and roots) from each pot were collected separately into paper bags, and dried at 80°C to constant weight. The dry weight of wheat plants was weighed using a high-precision electronic balance to finally obtain total biomass.

We obtained leaf cross-sections of the middle part of leaves on winter wheat with the paraffin section method of Sage et al (1995). The anatomical features of the leaves were observed and photographed under a microscopy and measured using Image J software (NIH, USA). We measured the thickness of the leaf mesophyll layer between the epidermal layers at five points in each cross-section. Twenty well-defined cells of the palisade layer and 20 cells of the spongy layer were randomly selected from each leaf cross-sectional image to measure cell length, cell width, cell area, and cell perimeter using the Auto CAD software.

### Measuring stomatal traits

Leaves of three winter wheat plants were randomly selected in each pot and stomatal imprints were collected by applying colorless and transparent nail varnish to the middle of the adaxial and abaxial for measuring the morphological traits of individual stoma during 10:00-12:00 am after 90-day treatments in the environmental growth chambers. The slides of stomatal imprints were placed under a photographic microscope for observation and photograph three microscopic fields were randomly selected and then four microscope photographs were taken from each field. We counted the number of stomata in each photograph and then calculated stomatal density on leaves of winter wheat, stomatal length, stomatal width, stomatal perimeter, stomatal area, and stomatal shape index were also measured and calculated with the AutoCAD 2010 software.

In this study, the center of stomatal openings on the surface of winter wheat leaves was used as the focal point to further determine the spatial distribution pattern of stomata on the leaves of winter wheat. The micrographs characterizing the distribution of stomata on the leaves were digitized in the same coordinate system by Arc GIS 10.0 software to obtain the spatial coordinate values of each stoma. Next, the point pattern analysis was estimated with Ripley’s K-function, a cumulative density function using the second moment of all point-to-point distances to evaluate two-dimensional distribution patterns at different scales (Xu, 2015; Zheng et al., 2020; Li et al., 2021). The results were plotted as Lhat(d) values, calculated as:


(1) $$Lhat\left(d\right)=\sqrt{\frac{K\left(d\right)}{\pi }}-d$$

where K(d), which represents the surface area of a circle with radius d, is the Ripley’s exponential function. When the pattern is Poisson random, the Lhat(d) is an expectation of zero for any value of d (Li et al., 2021). By executing a random distribution 100 times, we used the Monte Carlo simulation to estimate the 95% boundary. At a given scale of d, the Lhat(d) value for stomata randomly distributed on the leaf surface should be within the 95% boundary. If the Lhat(d) value is greater than the upper 95% boundary, the stomata follow a cluster distribution, otherwise, the stomata follow a regular distribution at the scale (Xu, 2015).

### Measuring leaf gas exchange

Five individual mature leaves (second fully expanded leaf from the top) were randomly selected from each treatment for measuring leaf gas exchange with a portable photosynthesis measurement system (LI-6400XT; LI-COR, USA) during 9:00-11:00 am after 90-day treatments. Leaves were firstly placed into the leaf chamber to determine the net photosynthetic rates (*P*_n_), stomatal conductance (*G*_s_), and transpiration rates (*T*_r_) at the light level of 1000 *μ*mol photon m^−2^ s^−1^ from a red-blue light source, which is the light saturation point for leaves of winter wheat. The temperature in the leaf chamber was set to 21°C (Control) or 26°C(warming) and the CO_2_ concentration was controlled at 400 *µ*mol mol^-1^ (*a*[CO_2_]) or 800 *µ*mol mol^-1^ (*e*[CO_2_]) during the leaf gas exchange measurements on winter wheat. We also calculated the leaf-level instantaneous water use efficiency (*WUEI*) with the ratio of leaf *P*_n_ and *T*_r_ (Zheng et al., 2020). Then, leaf dark respiration rate (*R*_d_) was also determined from the same leaves for measuring *P*_n_. After leaf *P*_n_ measurements, we turned off the red-blue light source in the leaf chamber of LI-6400XT, and then measured leaf *R*_d_ at the same temperature and CO_2_ concentration as the *P*_n_ measurements in the leaf chamber.

### Analyzing biochemical compositions

The dried leaves of winter wheat were ground to powders using a ball mill, and then analyzed the contents of glucose, fructose, sucrose, and starch according to the method of Hedrix et al. (1994). In addition to nonstructural carbohydrates, leaf total carbon (C) and nitrogen (N) were also determined with an elemental analyzer (VarioMax CN, Elementary Corp. Germany).

### Measuring enzyme activity and genes expression of Rubisco

The enzyme activity of Rubisco was measured by the method of Jiang et al. (2012). Moreover, we also analyzed the expression of Rubisco coding gene *RbcL3* and *RbcS2* according to the method of Livak et al. (2001). Specifically, total RNA was extracted using an RNA purification kit (Shanghai Shenggong Bioengineering Technology Service Co., Ltd.), and first-strand cDNA was synthesized according to the instructions of AMV first-strand cDNA synthesis kit (Shanghai Shenggong Bioengineering Technology Service Co., Ltd.). The primers for *RbcL3*, *RbcS2*, and *actin* were designed separately for amplification using Primer Premier 5.0 according to the sequences in the Genebank database. Specifically, the primer sequences for Rubisco key coding gene *RbcL3* were 5’-TAAATCACAGGCCGAAAC-3’ and 5’-GGCAATAATGAGCCAAAGT-3’. The primer sequence of *RbcS2* is 5’-AGCAACGGCGGAAGGAT-3’ and 5’-GCTCACGGAAGACGAAACC-3’. Subsequently, the expression of Rubisco coding gene was determined using a fluorescent quantitative PCR instrument.

### Statistical analysis

In the current study, we used a split-plot experimental design with three factors: water deficiency, [CO_2_] and warming. The split-plot three-way ANOVA was used to test the main effects of water deficiency, [CO_2_] and warming on plant biomass, stomatal traits, leaf gas exchanges, and biochemical compositions of winter wheat. The homogeneity and normal distribution of variances assumptions were evaluated before we ran the ANOVA analysis, and all of our data passed the assumptions. Results were considered significant if *P ≤* 0.05. All statistical analyses were used SPSS 13.0 software (SPSS Inc., Chicago, IL, USA), with all graphs produced in sigmaplot 10.

## Results

### The main effects of water deficiency, *e*[CO_2_], and warming

**Figure 1:**
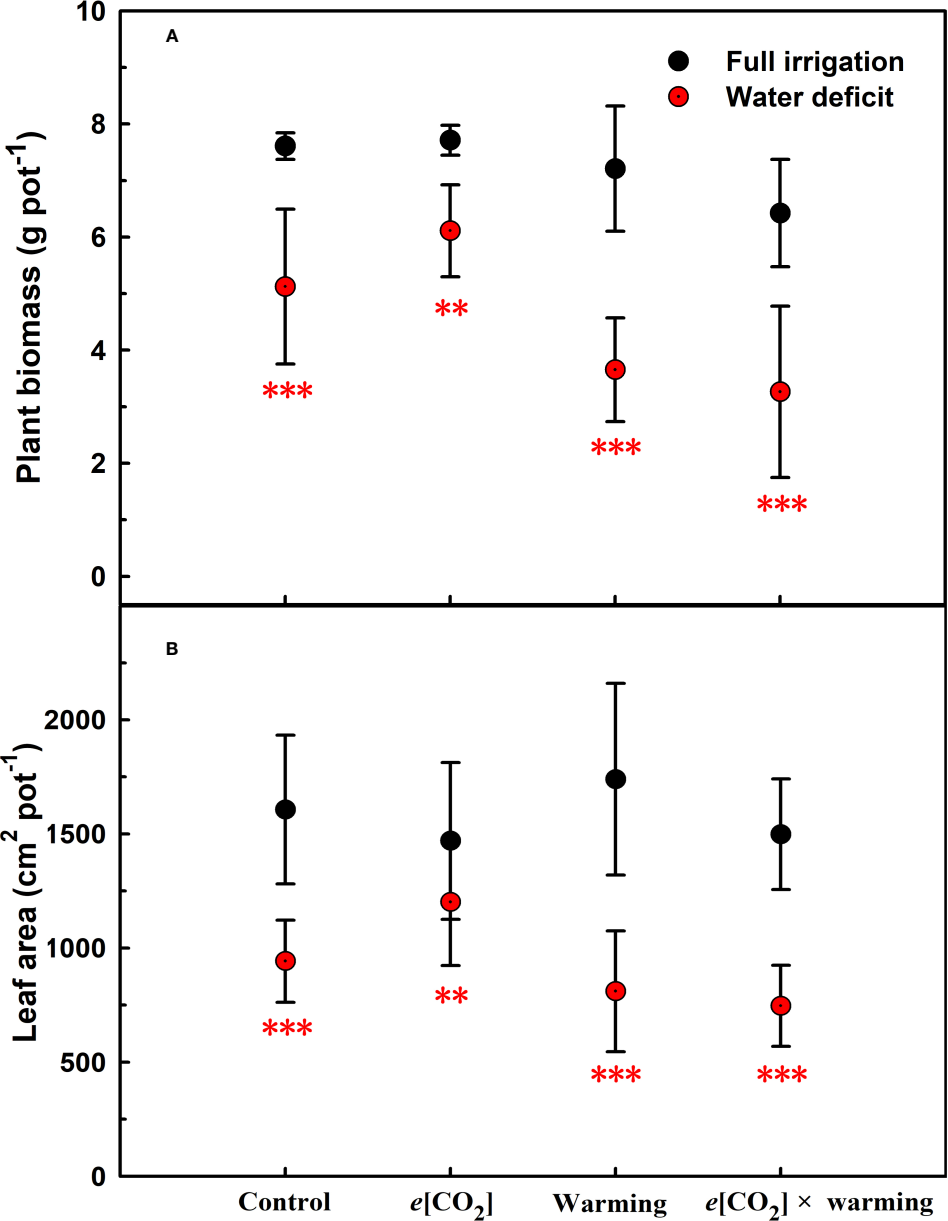
Effects of elevated [CO_2_] and experimental warming on the biomass **(A)** and leaf area **(B)** at different water conditions of winter wheat. Note that the black circle represents full irrigation, and the red circle represents water deficit. Values are means ± SD (n = 4). The symbol ** and *** indicate that the significant difference between full irrigation and water deficit under *e*[CO_2_], warming, and *e*[CO_2_] × warming are p<0.01 and p<0.001, respectively.

**Figure 2:**
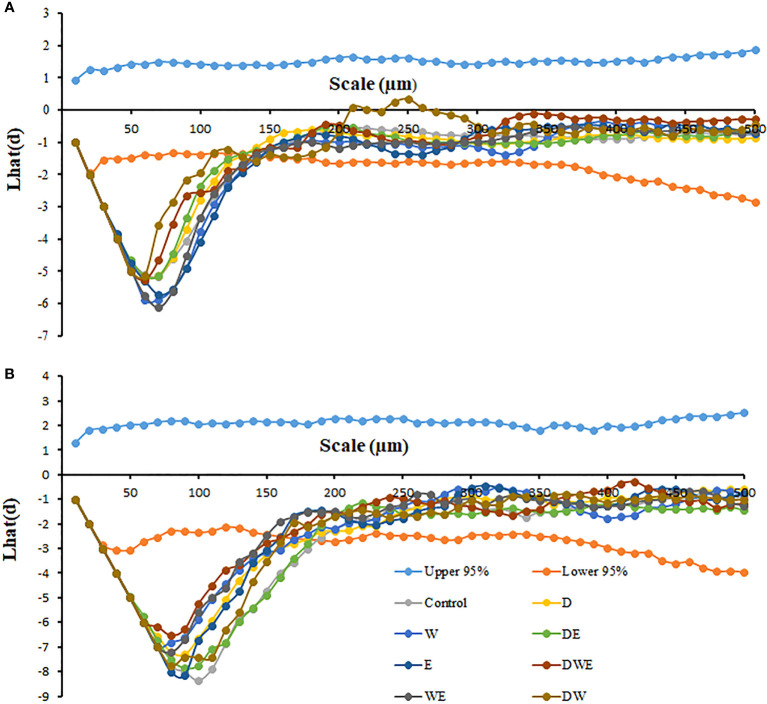
Effects of elevated [CO_2_] and experimental warming on the leaf spatial distribution pattern of stomata on the adaxial **(A)** and abaxial **(B)** surfaces at different water conditions of winter wheat. (The upper 95% means the upper boundary of the 95% confidence envelope, the lower 95% means the lower boundary of the 95% confidence envelope. The Lhat(d) value is the nearest neighbor distance, and stomata follow a regular distribution at the scale when the Lhat(d) value is lower than the 95% boundary with the smaller the minimum Lhat(d) value, the more regular spatial distribution pattern of stomata).

**Figure 3:**
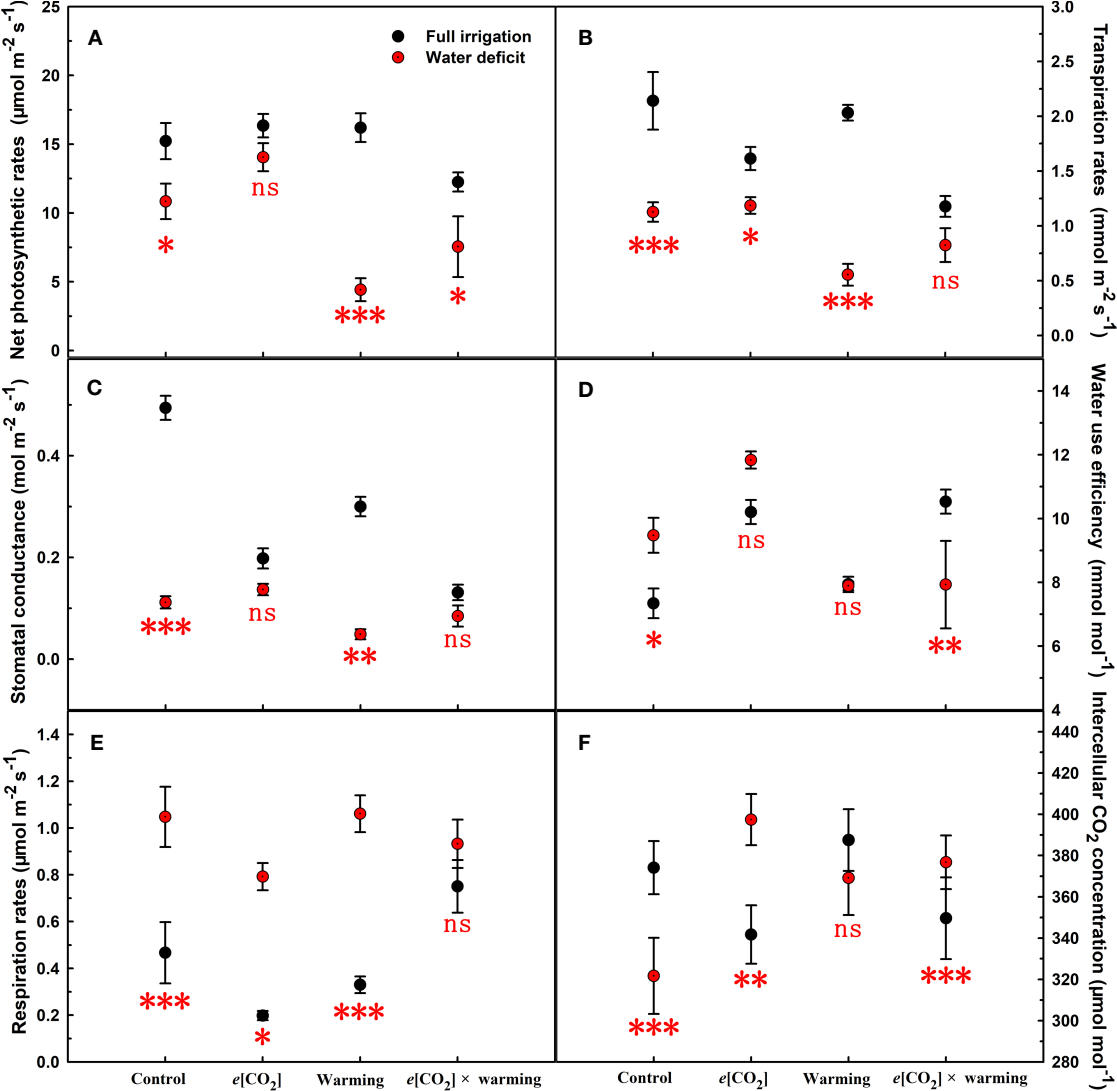
Responses of leaf net photosynthetic rates **(A)**, transpiration rates **(B)**, stomatal conductance **(C)**, water use efficiency **(D)**, respiration rates **(E)**, and intercellular CO_2_ concentration **(F)** to elevated [CO_2_] and experimental warming at different water conditions of winter wheat. Note that the black circle represents full irrigation, and the red circle represents water deficit. Values are means ± SD (n = 4). The symbol *, **, and *** indicate that the significant difference between full irrigation and water deficit under *e*[CO_2_], warming, and *e*[CO_2_] × warming are p<0.05, p<0.01, and p<0.001, respectively; ns denote no significant differences in full irrigation and water deficit at 0.05 level.

**Figure 4:**
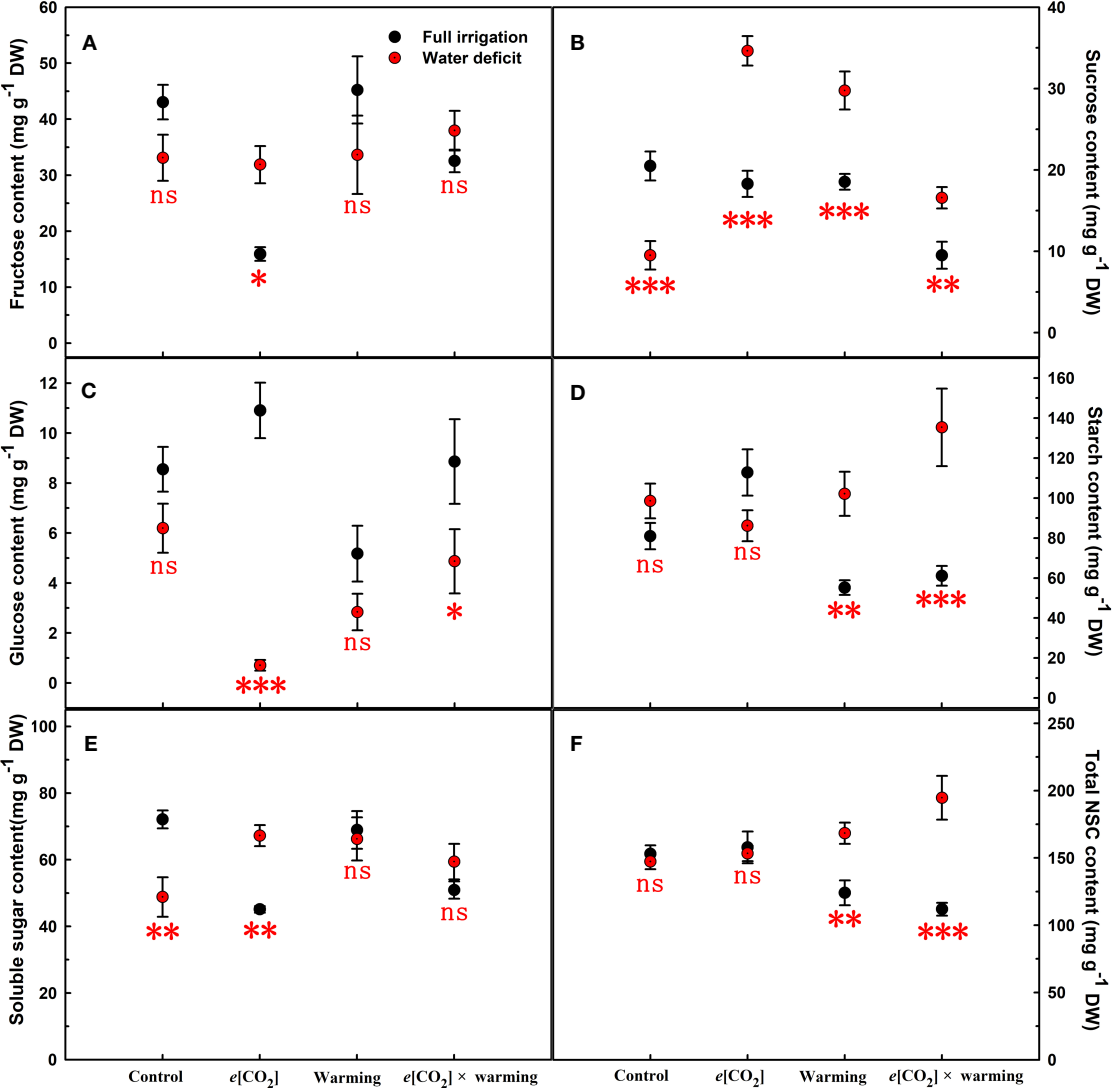
Effects of elevated [CO_2_] and experimental warming on fructose **(A)**, sucrose **(B)**, glucose **(C)**, starch **(D)**, soluble sugar **(E)**, and total NSC **(F)** at different water conditions of winter wheat. Note that the black circle represents full irrigation, and the red circle represents water deficit. Values are means ± SD (n = 4). The symbol *, **, and *** indicate that the significant difference between full irrigation and water deficit under *e*[CO_2_], warming, and *e*[CO_2_] × warming are p<0.05, p<0.01, and p<0.001, respectively; ns denote no significant differences in full irrigation and water deficit at 0.05 level.

**Figure 5:**
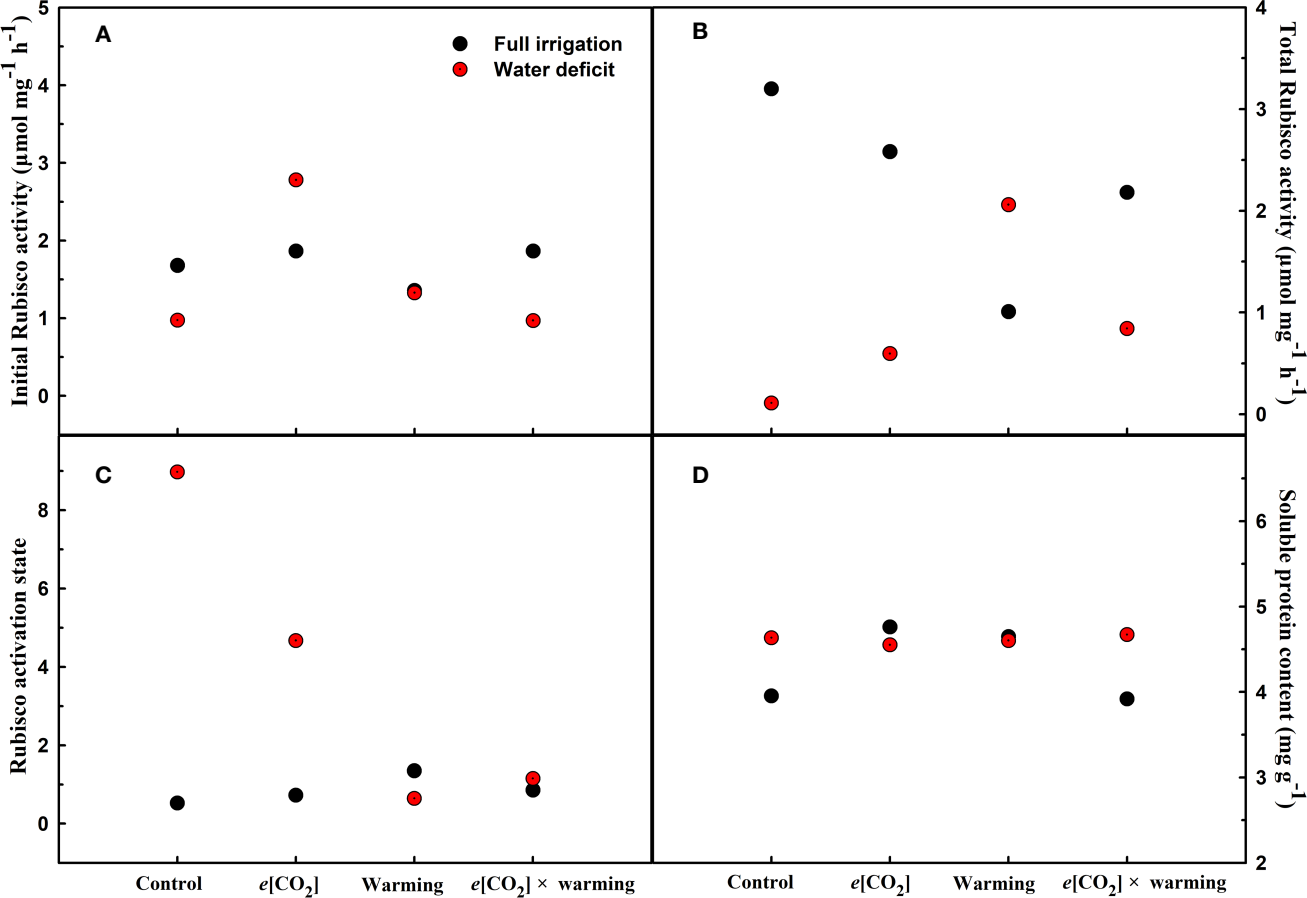
Effects of elevated [CO_2_] and experimental warming on initial Rubisco activity **(A)**, total Rubisco activity **(B)**, Rubisco activity state **(C)**, and soluble protein **(D)** at different water conditions of winter wheat. Note that the black circle represents full irrigation, and the red circle represents water deficit.

We found negative impacts of soil water deficiency on winter wheat, where the plant biomass and leaf area were decreased by net photosynthetic rate. Furthermore, water deficiency treatment (D) had negative effects on some morphological traits, such as total biomass and leaf areas, stomatal parameters, and spatial distribution pattern of winter wheat, more specifically D decreased total biomass and leaf areas (all *p*<0.001) and stomatal regular patterns on abaxial of winter wheat (**Table 1**; **Figures 1**, **2B**). By contrast, water deficiency significantly increased the stomatal width (*p*<0.001), stomatal area (*p*<0.001), and stomatal shape index (SSI) (*p*<0.001) on the adaxial leaf surface (**Tables 2**, **3**). Moreover, water deficiency substantially decreased the net photosynthetic rates (*P*_n_), transpiration rates (*T*_r_), stomatal conductance (*G*_s_) (all *p*<0.001), and intercellular CO_2_ concentration (*C*_i_) (*p=*0.046), while drastically increased the leaf dark respiration rates (*R*_d_) of winter wheat (*p*<0.001; **Figure 3**; **Table 4**). Water deficiency significantly reduced the total soluble sugar content of winter wheat (p<0.05), which was mainly due to a significant decline in sucrose content (p<0.05; **Figure 4**). Furthermore, water deficiency enhanced the Rubisco activation state and soluble protein content (**Figure 5**), but the initial Rubisco activity and total Rubisco activity were markedly decreased by water deficiency. In addition, water deficiency significantly increased the mesophyll cell perimeter (*p*=0.002), mesophyll cell area (*p*=0.006), mesophyll cell length (*p*=0.002), and leaf thickness (*p*<0.001; **Table 7**).

**Table 1:** Interactive effects of elevated [CO_2_] and experimental warming on the biomass and leaf area parameters at different water conditions of winter wheat.

Treatments	Total biomass	Leaf area
[CO_2_]	*p*=0.940	*p*=0.347
Water	***p*<0.001**	***p*<0.001**
Warming	***p*<0.001**	***p*=0.030**
[CO_2_] × water	*p*=0.272	***p*=0.004**
[CO_2_] × warming	*p*=0.055	***p*=0.028**
Water × warming	***p*=0.027**	***p*<0.001**
[CO_2_] × water × warming	*p*=0.674	*p*=0.260

Note that the bold values indicate a significant effect on the indicators.

**Table 2:** Effects of elevated [CO_2_] and experimental warming on the stomatal density and morphological traits of individual stomata at different water conditions of winter wheat.

Parameters	Leaf surfaces	Full irrigation				Water deficiency				*p*-values
		Control	*e*[CO_2_]	Warming	*e*[CO_2_] × warming	Drought	*e*[CO_2_]	Warming	*e*[CO_2_] × warming	
Stomatal density (SD No. mm^-2^)	adaxial	29.2 ± 3.5	40.3 ± 8.1	38.5 ± 9.1	45.0 ± 10.1	32.5 ± 3.3	34.7 ± 3.4	38.5 ± 6.0	42.8 ± 4.5	***p*<0.001**
	abaxial	51.1 ± 5.4	64.8 ± 9.3	55.7 ± 6.1	60.4 ± 7.9	61.2 ± 5.4	60.1 ± 8.9	56.4 ± 7.6	47.6 ± 10.6	***p*<0.001**
Stomatal length (SL μm)	adaxial	40.4 ± 4.0	38.0 ± 1.8	40.7 ± 3.1	38.9 ± 4.2	38.9 ± 5.2	40.3 ± 4.2	32.9 ± 4.7	38.1 ± 1.8	***p*=0.001**
	abaxial	37.0 ± 3.5	39.2 ± 6.0	39.1 ± 2.2	34.8 ± 1.8	38.9 ± 3.2	42.5 ± 2.6	34.9 ± 3.6	37.3 ± 2.4	***p*=0.001**
Stomatal width (SW μm)	adaxial	4.1 ± 0.7	3.6 ± 0.5	5.1 ± 0.5	4.3 ± 0.4	4.7 ± 0.4	3.5 ± 0.1	3.4 ± 0.5	3.7 ± 0.9	***p*<0.001**
	abaxial	4.0 ± 1.3	4.1 ± 1.0	4.4 ± 0.3	3.9 ± 0.2	4.3 ± 0.5	3.5 ± 0.3	3.1 ± 0.6	3.4 ± 0.8	*p*=0.070
Stomatal perimeter (SP μm)	adaxial	85.2 ± 8.7	79.3 ± 4.0	86.6 ± 6.4	81.8 ± 8.5	82.5 ± 10.1	83.9 ± 8.4	69.9 ± 8.6	79.4 ± 6.4	***p*=0.001**
	abaxial	78.3 ± 8.3	83.1 ± 13.6	82.7 ± 4.3	73.4 ± 4.2	82.3 ± 6.6	88.8 ± 4.9	72.8 ± 8.4	78.6 ± 4.9	***p*=0.011**
Stomatal area (SA μm^2^)	adaxial	167.4 ± 54.0	129.5 ± 21.1	211.1 ± 29.2	162.8 ± 22.3	191.4 ± 34.3	129.1 ± 18.7	102.4 ± 30.3	122.9 ± 37.9	***p*<0.001**
	abaxial	146.4 ± 66.5	171.2 ± 68.2	174.1 ± 17.0	138.6 ± 16.1	167.9 ± 26.7	146.9 ± 10.2	111.5 ± 36.4	121.7 ± 26.2	*p*=0.123
Stomatal shape Index (SSI)	adaxial	0.15 ± 0.01	0.14 ± 0.01	0.17 ± 0.01	0.16 ± 0.01	0.17 ± 0.01	0.14 ± 0.01	0.14 ± 0.01	0.14 ± 0.01	***p*<0.001**
	abaxial	0.15 ± 0.02	0.15 ± 0.01	0.16 ± 0.01	0.16 ± 0.01	0.16 ± 0.01	0.14 ± 0.01	0.14 ± 0.01	0.14 ± 0.01	***p*=0.008**

Note that the bold values indicate a significant effect on the indicators.

**Table 3:** Interactive effects of elevated [CO_2_] and experimental warming on stomatal parameters at different water conditions of winter wheat.

Stomatal traits	SD	SL	SW	SP	SA	SSI
[CO_2_]	***p*<0.001**	*p*=0.180	***p*<0.001**	*p*=0.402	***p*=0.003**	***p*<0.001**
Water	*p*=0.138	*p*=0.337	***p*<0.001**	*p*=0.209	***p*<0.001**	***p*<0.001**
Warming	*p*=0.142	***p*<0.001**	*p*=0.120	***p*<0.001**	***p*=0.030**	*p*=0.313
Leaf surface	***p*<0.001**	*p*=0.360	*p*=0.156	*p*=0.404	*p*=0.462	*p*=0.881
[CO_2_] × water	***p*<0.001**	***p*<0.001**	*p*=0.940	***p*<0.001**	*p*=0.394	***p*<0.001**
[CO_2_] × warming	***p*=0.011**	*p*=0.479	*p*=0.167	*p*=0.553	*p*=0.405	*p*=0.011
[CO_2_] × leaf surface	***p*=0.042**	*p*=0.724	***p*=0.026**	*p*=0.427	***p*=0.027**	***p*=0.006**
Water × warming	***p*=0.025**	***P*=0.001**	***p*<0.001**	***p*=0.001**	***p*<0.001**	***p*<0.001**
Water × leaf surface	*p*=0.777	***p*=0.018**	*p*=0.845	***p*=0.029**	*p*=0.414	*p*=0.244
Warming × leaf surface	***p*<0.001**	*p*=0.343	*p*=0.221	*p*=0.227	*p*=0.148	*p*=0.824
[CO_2_] × water × warming	*p*=0.297	*P*=0.069	***p*<0.001**	***p*=0.040**	***p*<0.001**	***p*<0.001**
[CO_2_] × water × leaf surface	***p*=0.022**	*p*=0.544	*p*=0.740	*p*=0.608	*p*=0.336	*p*=0.579
[CO_2_] × warming × leaf surface	*p*=0.059	***p*=0.009**	*p*=0.433	***p*=0.016**	***p*=0.034**	*p*=0.622
Water × warming × leaf surface	***p*=0.019**	*p*=0.565	***p*=0.020**	*p*=0.483	***p*=0.046**	*p*=0.051
[CO_2_] × water × warming × leaf surface	*p*=0.450	*p*=0.605	*p*=0.837	*p*=0.505	*p*=0.984	*p*=0.557

Note that the bold values indicate a significant effect on the indicators.

**Table 4:** Interactive effects of elevated [CO_2_] and experimental warming on photosynthesis parameters at different water conditions of winter wheat.

Treatments	*P*_n_	*T*_r_	*G*_s_	*WUE*	*R*_d_	*C*_i_
[CO_2_]	*p*=0.361	***p*=0.014**	***p*=0.008**	***p*<0.001**	*p*=0.946	*p*=0.917
Water	***p*<0.001**	***p*<0.001**	***p*<0.001**	*p*=0.551	***p*<0.001**	***p*=0.046**
Warming	***p*<0.001**	***p*=0.001**	***p*=0.013**	***p*=0.018**	*p*=0.257	***p*<0.001**
[CO_2_] × water	***p*=0.021**	***p*<0.001**	***p*=0.001**	*p*=0.108	***p*=0.007**	***p*<0.001**
[CO_2_] × warming	*p*=0.185	*p*=0.774	*p*=0.343	*p*=0.170	***p*=0.049**	*p*=0.091
Water × warming	***p*=0.014**	*p*=0.347	*p*=0.317	***p*=0.001**	*p*=0.967	***p*<0.001**
[CO_2_] × water ×warming	*p*=0.197	*p*=0.195	*p*=0.423	*p*=0.274	*p*=0.268	*p*=0.081

Note that the bold values indicate a significant effect on the indicators.

**Figure 6:**
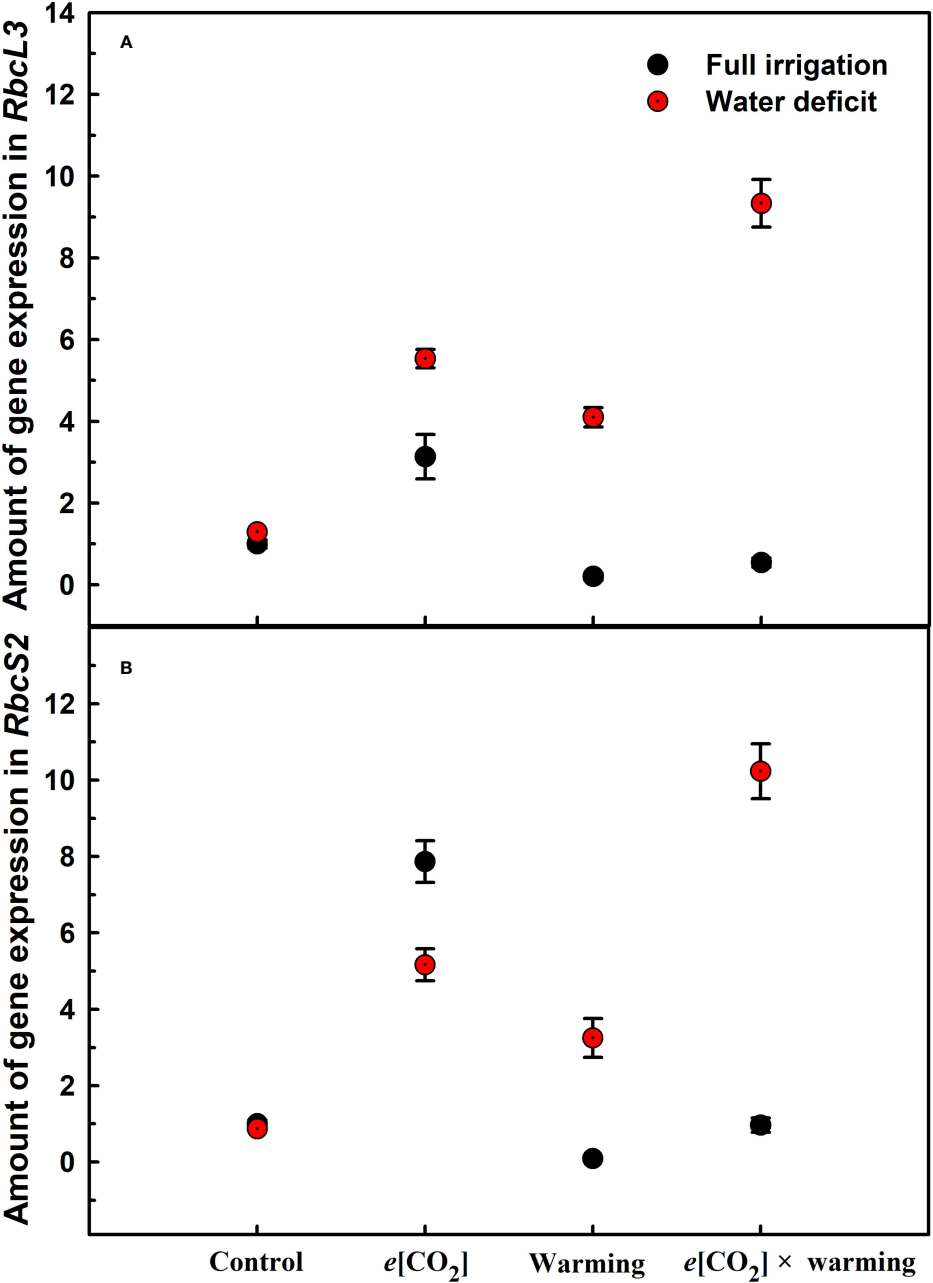
Effects of elevated [CO_2_] and experimental warming on the amount of gene expression in RbcL3 **(A)** and RbcS2 **(B)** of Rubisco at different water conditions in winter wheat leaves. Note that the black circle represents full irrigation, and the red circle represents water deficit.

**Figure 7:**
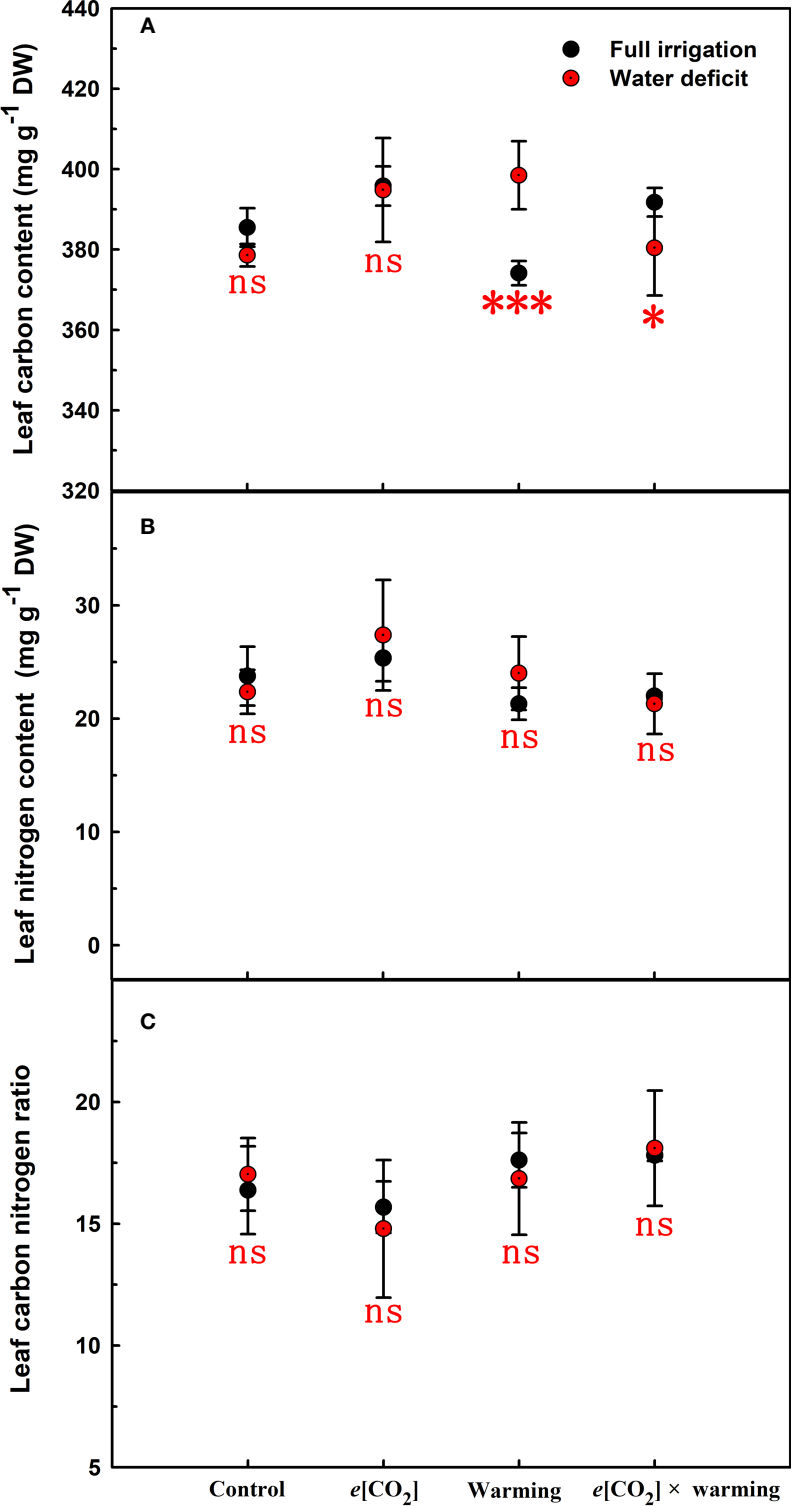
Effects of elevated [CO_2_] and experimental warming on leaf carbon **(A)**, nitrogen **(B)**, and carbon nitrogen ratio **(C)** at different water conditions of winter wheat. Note that the black circle represents full irrigation, and the red circle represents water deficit. Values are means ± SD (n = 4). The symbol * and *** indicate that the significant difference between full irrigation and water deficit under *e*[CO_2_], warming, and *e*[CO_2_] × warming are p<0.05, and p<0.001, respectively; ns denote no significant differences in full irrigation and water deficit at 0.05 level.

Elevated CO_2_ concentration (*e*[CO_2_]) increased stomatal density on both leaf surfaces (all *p*<0.001) as well as enhanced the stomatal area on the abaxial leaf surface (*p*=0.003) and the regularity of stomatal distribution on the adaxial leaf surface (**Figure 2A**), whereas decreased the stomatal width (*p*<0.001), stomatal area (*p*=0.003) and stomatal shape index (*p*<0.001) on the adaxial surface of leaves (**Tables 2**, **3**). Furthermore, *e*[CO_2_] substantially decreased leaf *G*_s_ (*p*=0.008) and *T*_r_ (*p*=0.014) mainly due to the declines of stomatal area. As a result, the leaf-level instantaneous water use efficiency (*WUEI*) was enhanced by *e*[CO_2_] (*p*<0.001; **Figure 3**; **Table 4**), although *P*_n_ was barely affected under *e*[CO_2_] (*p*>0.05; **Figure 3**; **Table 4**). Moreover, the soluble sugar content was significantly reduced (*p*=0.015) under *e*[CO_2_], mainly attributed to the decrease in fructose content (*p*=0.005), but *e*[CO_2_] marginally increased the leaf carbon content (*p*=0.017; **Figures 4**, **7**; **Tables 5**, **8**). Meanwhile, Rubisco activation state and soluble protein content of winter wheat under *e*[CO_2_] was obviously higher than control (all *p*<0.05; **Figure 5**) due to the increase of the amount of gene expression in *RbcL3* and *RbcS2* (all *p*<0.001; **Figure 6**; **Table 6**). In terms of the anatomical traits, *e*[CO_2_] also increased mesophyll cell length, but drastically decreased the leaf thickness (all *p*<0.001; **Table 7**).

**Table 5:** Interactive effects of elevated [CO_2_] and experimental warming on nonstructural carbohydrates at different water conditions of winter wheat.

Treatments	Fructose	Sucrose	Glucose	Soluble sugar	Starch	TNC
[CO_2_]	***p*=0.005**	*p*=0.871	*p*=0.406	***p*=0.015**	*p*=0.056	*p*=0.345
Water	*p*=0.992	***p*<0.001**	***p*<0.001**	*p*=0.719	***p*=0.001**	***p*<0.001**
Warming	***p*=0.042**	*p*=0.067	*p*=0.114	*p*=0.346	*p*=0.405	*p*=0.639
[CO_2_] × water	***p*=0.001**	***p*<0.001**	***p*=0.005**	***p*<0.001**	*p*=0.566	*p*=0.142
[CO_2_] × warming	*p*=0.105	***p*<0.001**	***p*=0.008**	*p*=0.214	*p*=0.507	*p*=0.900
Water × warming	*p*=0.314	***p*=0.008**	*p*=0.053	*p*=0.590	***p*<0.001**	***p*<0.001**
[CO_2_] × water ×warming	*p*=0.459	***p*<0.001**	*p*=0.054	***p*=0.013**	***p*=0.021**	*p*=0.165

Note that the bold values indicate a significant effect on the indicators.

**Table 6:** Interactive effects of elevated [CO_2_] and experimental warming on the amount of gene expression of Rubisco at different water conditions of winter wheat.

Treatments	*RbcL3*	*RbcS2*
[CO_2_]	***p*<0.001**	***p*<0.001**
Water	***p*<0.001**	***p*<0.001**
Warming	***p*<0.001**	*p*=0.624
[CO_2_]×water	***p*<0.001**	***p*<0.001**
[CO_2_]×warming	*p*=0.178	***p*<0.001**
Water×warming	***p*<0.001**	***p*<0.001**
[CO_2_]×water×warming	***p*<0.001**	***p*<0.001**

Note that the bold values indicate a significant effect on the indicators.

**Table 7:** Effects of elevated CO_2_ concentration and experimental warming on leaf mesophyll cells at different water conditions of winter wheat.

Parameters		Cell length	Cell width	Cell perimeter	Cell area	Leaf thickness
Full irrigation	Control	22.45 ± 2.10	17.47 ± 1.68	66.97 ± 6.65	332.16 ± 65.1	132.04 ± 9.00
	*e*[CO_2_]	24.06 ± 2.54	15.94 ± 2.41	62.41 ± 8.97	286.88 ± 80.10	121.04 ± 5.83
	Warming	20.78 ± 0.99	14.94 ± 1.28	64.13 ± 4.54	294.31 ± 37.78	102.10 ± 4.51
	*e*[CO_2_]×Warming	22.23 ± 2.24	15.53 ± 1.22	63.01 ± 6.10	290.31 ± 61.50	125.67 ± 10.21
Water deficiency	Drought	24.18 ± 1.73	17.14 ± 1.7	71.51 ± 6.21	360.21 ± 54.40	157.60 ± 9.96
	*e*[CO_2_]	26.66 ± 2.50	17.84 ± 1.94	77.16 ± 7.46	418.73 ± 75.31	130.62 ± 11.29
	Warming	20.60 ± 1.15	14.54 ± 1.05	57.20 ± 3.55	236.72 ± 29.88	148.83 ± 9.70
	*e*[CO_2_]×Warming	22.83 ± 2.37	15.92 ± 1.61	66.02 ± 5.30	313.16 ± 46.93	138.06 ± 8.98
[CO_2_]		***p<*0.001**	*p*=0.358	*p*=0.066	*p*=0.056	***p<*0.001**
Water		***p*=0.002**	*p*=0.214	***p*=0.002**	***p*=0.006**	***p<*0.001**
Warming		***p<*0.001**	***p<*0.001**	***p<*0.001**	***p<*0.001**	***p<*0.001**
[CO_2_]×water		*p*=0.281	***p*=0.017**	***p<*0.001**	***p<*0.001**	***p<*0.001**
[CO_2_]×warming		*p*=0.788	***p*=0.025**	*p*=0.166	*p*=0.185	***p<*0.001**
Water×warming		***p*=0.012**	*p*=0.207	***p<*0.001**	***p<*0.001**	***p<*0.001**
[CO_2_]×water×warming		*p*=0.955	*p*=0.245	*p*=0.956	*p*=0.599	***p<*0.001**

Note that the bold values indicate a significant effect on the indicators.

**Table 8:** Interactive effects of elevated [CO_2_] and experimental warming on leaf carbon (C) and nitrogen (N).

Treatments	Carbon	Nitrogen	C/N ratio
[CO_2_]	***p*=0.017**	*p*=0.205	*p*=0.552
Water	*p*=0.627	*p*=0.461	*p*=0.776
Warming	*p*=0.343	***p*=0.007**	***p*=0.013**
[CO_2_] × water	***p*=0.007**	*p*=0.994	*p*=0.845
[CO_2_] × warming	***p*=0.014**	***p*=0.021**	*p*=0.087
Water × warming	*p*=0.051	*p*=0.702	*p*=0.924
[CO_2_] × water × warming	***p<*0.001**	*p*=0.064	*p*=0.306

Note that the bold values indicate a significant effect on the indicators.

Our results also showed that experimental warming increased the leaf area (*p*=0.030), stomatal length (*p*<0.001), stomatal perimeter (*p*<0.001), and stomatal area (*p*=0.030), but decreased the total plant biomass of winter wheat (*p*<0.001; **Figure 1**; **Tables 1**–**3**). Moreover, the stomatal distribution regularity on the adaxial surface was enhanced by experimental warming (**Figure 2**). Similarly, experimental warming increased the Pn (*p*<0.001), WUE (*p*=0.018), and Ci (*p*<0.001), while decreased the Tr (*p*<0.001), Gs (*p*=0.013), and glucose content (*p*=0.042; **Figures 3**, **4**; **Tables 4**, **5**). The mesophyll cell length, cell width, cell perimeter, cell area, and leaf thickness were also magically reduced under experimental warming (all *p*<0.001; **Table 7**).

### The interactive effects of water deficiency and experimental warming

Experimental warming had a negative impact on plant biomass (*p*=0.027), leaf area under water deficiency (all *p*<0.001; **Figure 1**; **Table 1**). Meanwhile, the stomatal density (*p*=0.025), stomatal length (*p*=0.001), stomatal width (*p*<0.001), stomatal perimeter (*p*=0.001), stomatal area (*p*<0.001), and stomatal shape index (*p*<0.001) as well as the regularity of abaxial stomatal spatial distribution pattern were also reduced under experimental warming and water deficiency (**Figure 2**; **Tables 2**, **3**). Similarly, we also found negatively interactive effects of experimental warming and water deficiency on leaf *P*_n_ (*p*=0.014), *WUE* (*p*=0.001), and Rubisco activation state (**Figure 5**) of wheat plants, although positive effects were observed on leaf sucrose (*p*=0.008), starch (*p*<0.001), TNC (*p*<0.001), and the amount of gene expression in *RbcL3* (*p*<0.001) and *RbcS2* (*p*<0.001) under experimental warming and water deficiency condition. Moreover, experimental warming significantly decreased the mesophyll cell length (*p*=0.012), cell perimeter (*p*<0.001), cell area (*p*<0.001), and leaf thickness (*p*<0.001) under water deficiency (**Table 7**).

### The interactive effects of soil water deficiency and elevated CO_2_ concentration

Our three-way ANOVA results showed interactive effects of *e*[CO_2_] and water deficiency on the morphological traits of leaves and stomata (all *p*<0.05). We found that *e*[CO_2_] significantly increased the leaf area of winter wheat under water deficiency (*p*=0.004; **Figure 1**; **Table 1**). Meanwhile, *e*[CO_2_] also increased the stomatal density on the adaxial leaf surface, and the stomatal length and perimeter on both leaf surfaces of winter wheat at water deficiency condition (all *p*<0.001; **Tables 2**, **3**). Moreover, *e*[CO_2_] substantially enhanced leaf *P*_n_, *T*_r_, and *G*_s_ by *c.* 30% (*p*=0.021), 50% (*p*<0.001), and 20% (*p*=0.001) under water deficiency, indicating the negative impacts of water deficiency on winter wheat might be partially mitigated by *e*[CO_2_], which could also be supported by the significantly interactive effects of *e*[CO_2_] and water deficiency on leaf *P*_n_ (*p*=0.021), *T*_r_ (*p*<0.001), and *G*_s_ (*p*=0.001) of winter wheat (**Table 4**). Furthermore, *e*[CO_2_] and water deficiency also interactively affected leaf carbon (*p*=0.007) and soluble sugar (*p*<0.001) with increasing leaf fructose (*p*=0.001) and sucrose (*p*<0.001). In addition, the expression amount of Rubisco coding genes were also significantly affected by the interactions of *e*[CO_2_] and water deficiency through enhancing the amount of gene expression in *RubcL3* (*p*<0.001; **Figure 6**; **Table 6**). Additionally, our ANOVA results also showed significantly interactive effects on the mesophyll cell width (*p*=0.017), cell perimeter (*p*<0.001), cell area (*p*<0.001), and leaf thickness under [CO_2_] × water (*p*<0.001; **Table 7**).

### The interactive effects of elevated CO_2_ concentration and experimental warming

Our results showed that *e*[CO_2_] significantly decreased the leaf area of winter wheat grown at experimental warming (*p*=0.028). However, the stomatal density and regularity of stomatal distribution pattern on the adaxial leaf surface was increased under *e*[CO_2_] and experimental warming conditions (**Figures 1**, **2**; **Tables 2**, **3**). Moreover, *e*[CO_2_] × warming reduced leaf sucrose content (*p*<0.001; **Figure 4**; **Table 5**), but dramatically increased leaf carbon (*p*=0.014; **Figure 7**; **Table 8**). Additionally, *e*[CO_2_] decreased total Rubisco activity, mesophyll cell width (*p*=0.025) and leaf thickness (*p*<0.001) under experimental warming (**Figure 5**; **Table 7**).

### The interactive effects of elevated CO_2_ concentration, water deficiency and experimental warming

We found significantly interactive effects of water deficiency, *e*[CO_2_], and experimental warming on the morphological traits of stomata such as stomatal width (*p*<0.001), stomatal perimeter (*p*=0.04), stomatal area (*p*<0.001), and stomatal shape index (*p*<0.001; **Table 3**). Meanwhile, the biochemical compositions in wheat leaves were also obviously changed under water deficiency, *e*[CO_2_], and experimental warming as shown by their interactions on leaf sucrose (*p*<0.001), starch (*p*=0.021), and soluble sugars (*p*=0.013; **Figure 4**; **Table 5**) as well as leaf carbon (*p*<0.001; **Figure 7**; **Table 8**). In addition to stomata and biochemical compositions, the expression amount of Rubisco coding gene *RbcL3* and *RbcS2* as well as leaf thickness were also interactively affected by water deficiency, *e*[CO_2_], and experimental warming (all *p*<0.001).

## Discussion

### The interactive effects of water deficiency and elevated [CO_2_]

The reduction in biomass and photosynthetic rate under water deficiency may be due to stomatal limitation or non-stomatal limitation (Yu et al., 2012; Wang et al., 2017). The former is typically caused by stomatal number, stomatal distribution pattern, and stomatal opening (Fan et al., 2020; Zheng et al., 2020), while the latter is attributed to metabolic disorders such as imbalance of carbon sink and source or reduced carboxylation efficiency due to reduced Rubisco activity (Parry et al., 2003; Aranjuelo et al., 2011). In the current study, we found a *c.*29% decrease in photosynthesis under water deficiency, mainly due to a significant *c.*77% decrease in stomatal conductance, and the increase in starch that inhibited the reduction phase of the Calvin cycle (**Figures 3**, **4**; **Tables 4**, **5**) (Sheen, 1990). Significantly lower *G*_s_ and *C*_i_ under water stress suggest that carboxylation efficiency may be severely restricted by water deficiency, as reported by others (Flexas et al., 2004; Yu et al., 2012).

Previous studies found that elevated [CO_2_] had a strong “fertilization effect” on C_3_ plants because the current environment did not reach the optimal CO_2_ concentration for photosynthetic rates (Xu, 2015; Zheng et al., 2018), while Högy et al. (2013) showed that increasing CO_2_ concentration by 150 μmol mol^-1^ barely affects the growth and development of winter wheat. In the present study, we found elevated [CO_2_] from 400 μmol mol^-1^ to 800 μmol mol^-1^ also did not affect biomass and *P*_n_ of winter wheat may be due to the decrease of *G*_s_. Nevertheless, elevated [CO_2_] did not interact with water deficiency on plant biomass, elevated [CO_2_] alleviated *P*_n_ in winter wheat under drought treatment, which was related to Gs, metabolic activity, and anatomical structure. Results showed that under *e*[CO_2_] × water, the reduction of *T*_r_ could suppress water loss in winter wheat to ensure normal metabolism. Additionally, the study found that elevated [CO_2_] allowed more photosynthetic products to be stored in the form of soluble sugars when winter wheat plants were subjected to water deficiency (**Figure 3**; **Table 4**), which may indicate that elevated CO_2_ concentrations from 400 μmol mol^-1^ to 800 μmol mol^-1^ favored Rubisco carboxylation rather than RuBP regeneration (Xu, 2015) may also partially explain that the biomass was barely affected (Wong, 1990; Zheng et al., 2018). Meanwhile, the nitrogen content in the leaves was not limited by elevated [CO_2_] and water deficiency, which ensured photosynthesis (Zong and Shangguan, 2016). Furthermore, we also found elevated [CO_2_] concentrations alleviated photosynthesis probably associated with elevated Rubisco activity as well as the amount of gene expression in *RbcL3* and *RbcS2* when plant under water deficiency (Parry et al., 2003; Hou et al., 2021). In addition to physiological factors, under water deficiency conditions, elevated [CO_2_] significantly enlarged the mesophyll cell area, which largely determined photosynthesis, as more chloroplasts could be accommodated (Zheng et al., 2018).

Previous results showed that drought significantly increased plant *R*_d_ *c*.24%, which may result in greater carbohydrate consumption (Yu et al., 2012), while *R*_d_ decreased by 20% by doubling ambient [CO_2_] (Drake et al., 1997). Our study found that under water deficiency, the respiration rate was downregulated by 24.4% at elevated [CO_2_], which facilitated the development of drought tolerance in winter wheat and thus stored more carbohydrates.

### The interactive effect of experimental warming and elevated [CO_2_]

Several studies have shown that experimental warming may promote plant growth by increasing photosynthesis in leaves while inhibiting plant growth at high temperatures (Hatfield et al., 2011). For example, Liu et al. (2020) showed that maize significantly increased *P*_n_, but decreased total biomass at 31°C, mainly because its growth temperature was still at its optimal temperature and the increase in Rubisco activity due to warming while *P*_n_ and total biomass decreased at 37°C, probably due to the accumulation of potent reactive oxygen species (ROS) at high temperature (Chen et al., 2022). The interactive effects of temperature and elevated [CO_2_] on plant growth are complex and tend to be multivariate (Liu et al., 2020; Yu et al., 2012; Duan et al., 2019a). For instance, Yu et al. (2012) found that elevated [CO_2_] mitigated the negative effects of experimental warming (30°C) on tall fescue by reducing Gs (20%), Rd (7%). However, Liu et al. (2020) concluded that elevated [CO_2_] could not alleviate *P*_n_ and total biomass of maize under experimental warming (31°C), while significantly enhanced plants *P*_n_ under high temperature stress (37°C). In the present study, the growth temperature of winter wheat was raised from 21 to 26°C, and plant biomass was barely affected by the experimental warming, which was directly supported by the slight variation in leaf photosynthesis. Interestingly, the significant decline in plant biomass of winter wheat due to *e*[CO_2_] × warming implies that wheat may be more affected under future climate change with warming and elevated [CO_2_] (Yu et al., 2012; Duan et al., 2019a; Wang et al., 2021). Our results showed that the decrease in plant photosynthesis (19.5%) was associated with the decrease in *G*_s_ (73%) and mesophyll cell area (12.6%) and the increase in C/N (10%), although the increase in WUE was due to a decrease in transpiration. Many studies found that elevated [CO_2_] reduced soluble protein content and increased C/N of winter wheat, which is consistent with our results (Wang et al., 2013; Xu, 2015). And we observed that *e*[CO_2_] × warming significantly increased *R*_d_ in winter wheat, which was mainly from TNC depletion. Overall, these results suggest that CO_2_ fertilization effects can be reduced by warming, and therefore the risk of climate change to global wheat yields may be underestimated.

### The interactive effect of water deficiency experimental warming and elevated [CO_2_]

The interactive effect of drought and warming under ambient [CO_2_] is more detrimental for plant growth than stress alone, which is consistent with the study of Yu et al. (2012). Previous studies have shown that elevated [CO_2_] can alleviate water stress or heat stress (Jagadish et al., 2014; Xu, 2015; Duan et al., 2019a; Zheng et al., 2020), but our results show that the biomass and *P*_n_ of winter wheat further decreased and C/N increased under the combined effect of elevated [CO_2_], experimental warming and water deficiency, implying that the combination of long-term water deficiency and experimental warming resulted in irreversible physiological damage in winter wheat. Yu et al. (2012) concluded that elevated [CO_2_] further reduced tall fescue *F*_v_ *: F*_m_ (33%), *V*_cmax_ (8%), and *J*_max_ (13%) under the combined effect of experimental warming and water deficiency, suggesting that elevated [CO_2_] may have played an additional negative effect under water × warming. Furthermore, in the current study, we observed that osmoregulation is reduced when plants are subjected to stress and nonstructural carbohydrates are not properly converted into structural carbohydrates for plant growth. (Zheng et al., 2014). However, elevated [CO_2_] significantly increased amount of gene expression in *RbcL3* and *Rbcs2* under water × warming deficiency but slightly affected Rubisco activity of winter wheat, which may be due to the plants were subjected to irreversible combined stress caused by experimental warming and drought (Aranjuelo et al., 2005; Yu et al., 2012). Overall, plants adapt to stress by adjusting their growth, physiology, cellular and molecular activities (Ahuja et al., 2010). Since winter wheat lacks vernalization in the environmental growth chamber, we did not conduct further studies on the effect of *e*[CO_2_] × water × warming on crop yield. It is worth noting that the combined effects of *e*[CO_2_] × water × warming deficiency on growth, physiology, and molecular mechanisms of winter wheat may also be confounded with other factors such as nitrogen deposition, phosphorus deficiency and, ozone concentration. Therefore, to better predict the effects of climate change on wheat production, further multifactorial experimental studies are necessary to fully understand the mechanisms and processes between plant growth and environmental changes.

## Conclusions

We found that water deficiency and experimental warming decreased the leaf *P*_n_ and *G*_s_, but increased the nonstructural carbohydrates, and thus reduced the biomass of winter wheat. In addition, elevated [CO_2_] partially alleviated the stress in winter wheat at the molecular level but could not alleviate the irreversible damage to the plant caused by water deficiency × warming. Overall, our results suggest that the synergistic effects of elevated [CO_2_], warming, and water deficiency may reduce plant biomass and leaf photosynthesis, thereby the global grain yield of winter wheat may be reduced under future climate change.

## Data availability statement

The original contributions presented in the study are included in the article/supplementary material. Further inquiries can be directed to the corresponding authors.

## Author contributions

ZC, LH, YZL, and YT designed the study. ZC, YL, LL, CC, and YW performed the experiment. ZC, YL, LL, CC, and WS analyzed the data. ZC, LH, YZL and YT wrote the initial manuscript. All authors contributed to the article and submitted and approved the submitted section.
